# Acute acalculous cholecystitis following COVID-19 vaccination: a case report

**DOI:** 10.11604/pamj.2022.41.291.33047

**Published:** 2022-04-11

**Authors:** Fabien Mukonki Kyungu, Aude Mukonki Katumba, Hermann Luhavo Kamwira, Aime Vonda Mayikuli, Adou Mala, Manix Ilunga Banza, Haladou Mahaman Manirou, Fiston Bolanda Tosali, Philippe Cilundika Mulenga

**Affiliations:** 1 Unit of Occupational Medicine and Environmental Research, Department of Public Health, Faculty of Medicine, University of Lubumbashi, Lubumbashi, Democratic Republic of the Congo,; 2 Occupational Health and Safety Department, Kibali Gold Mine, Barrick Gold Corporation, Doko, Haut-Uélé Province, Democratic Republic of the Congo,; 3 Department of Public Health, Faculty of Medicine, University of Lubumbashi, Lubumbashi, Democratic Republic of the Congo,; 4 Institut National de Recherche Biomédicale, Goma, Province du Nord-Kivu, République Démocratique du Congo,; 5 Department of Surgery, Faculty of Medicine, University of Lubumbashi, Lubumbashi, Democratic Republic of the Congo,; 6 Occupational Health and Safety Department, Barrick Gold Corporation, Bamako, Mali,; 7 Centre de Traitement de COVID-19, Durba, Watsa, Province du Haut-Uélé, République Démocratique du Congo

**Keywords:** Acute acalculous cholecystitis, liver dysfunction, COVID-19 vaccination, case report

## Abstract

Acute acalculous cholecystitis is an acute inflammation of the gallbladder in the absence of stones, usually occurring in elderly and critically ill patients with underlying conditions. A 29-year-old man presented to the hospital complaining of abdominal pain in the right hypochondrium with permanent fever three days after Janssen COVID-19 vaccine inoculation. Abdominal ultrasound revealed a thickened gallbladder wall without evidence of gallstone consistent of an acute acalculous cholecystitis. Blood analyses revealed thrombocytopenia, eosinophilia and liver dysfunction. The Polymerase Chain Reaction (PCR) COVID-19 test was negative. As treatment, the patient benefited of pain management, antibiotic and fluid. In the evolution, there was a regression of clinical signs with persistence of liver dysfunction. The patient was discharged ten days after hospitalization. The Janssen COVID-19 vaccine is likely to induce acute acalculous cholecystitis as adverse event following vaccination.

## Introduction

Acute acalculous cholecystitis is characterized by acute inflammation of the gallbladder in the absence of stones, eldery and ill patients with underlying conditions are severely affected. This pathology may be insidious, with unexplained fever, leukocytosis, hyperamylasemia, or abnormal aminotransferases, and patients often lack right upper quadrant tenderness [1,2]. On February 27, 2021, the Food and Drug Administration (FDA) issued an Emergency Use Authorization (EUA) for the Janssen COVID-19 vaccine to prevent lung infection due to coronavirus (SARS-CoV-2) [3-5]. In United States from March 2^nd^ to April 21, 2021 about 7.98 million doses of the Janssen COVID-19 vaccine were administrated, rare cases of Cerebral Venous Sinus Thrombosis (CVST) with thrombocytopenia and Thrombosis with Thrombocytopenia Syndrome (TTS) were reported to the Vaccine Adverse Event Reporting System (VAERS) among vaccine recipients with diverse locations [5]. We present a case of acute acalculous cholecystitis following a single dose inoculation of Janssen COVID-19 vaccine observed to a young Congolese man.

## Patient and observation

**Patient information:** the patient was a 29-year-old man with history of a good physical health and no abdominal surgery or any chronic condition including inflammatory bowel disease who presented to the hospital with headache, nausea, high grade fever, right upper quadrant abdominal pain and passing dark colored urine after inoculation of Janssen COVID-19 vaccine.

**Clinical findings:** the clinical parameters were 93/min Heart rate, 153/121 mmHg blood arterial pressure, 20/min Respiratory rate, 95% spO_2_ and 39.2°C of temperature. The physical examination revealed a tenderness in the right hypochondrium (edge of the liver not well delimited because of the pain). The rest of the exam was unremarkable.

**Timeline of current episode:** the symptoms started two days ago after getting a single dose of Janssen COVID-19 vaccine on November 8^th^, 2021. He developed fever, fatigue and myalgia as adverse events for which he benefited of fluid, fever and pain management as treatment. The persistence of fever and appearance of abdominal pain motivated the referral at International Hospital of Kampala on November 11^th^, 2021.

**Figure 1:**
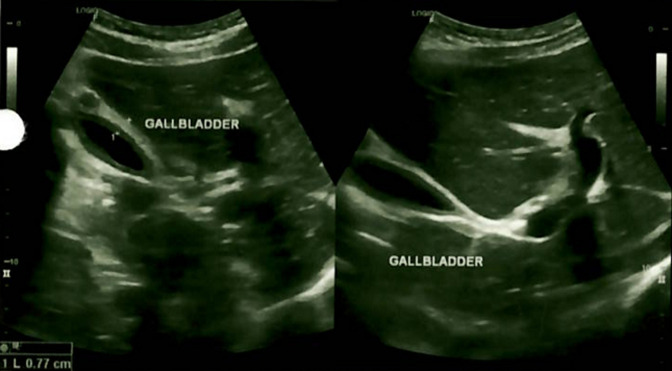
acute acalculous cholecystitis

**Diagnostic assessment:** urine and blood analyses, malaria thick smear, malaria antigen rapid test, hepatitis B surface antigen, HIV 1 and HIV 2 serological test, abdominal ultrasound and Polymerase Chain Reaction (PCR) COVID-19 test were performed. The urine colour was dark yellow, appearance turbid, with few leucocytes, epithelial cells and pus cells. On the multistix urine analyze, ketones+, leucocytes+ and trace of protein were found. The liver function was abnormal with ASP 493.9 IU/L (healthy adult male <38), ALT 244.7U/L (normal range 0-40), GGT 85U/L (normal range 5-40). The CRP was 148.62 mg/L (normal range <5). The full blood count showed thrombocytopenia 79.10^9^/L (normal range 140-440) and leucopenia 2.83. 10^9^/L (normal range 4-11). The renal function was normal, the malaria antigen rapid test, malaria thick smear, hepatitis B surface antigen, HIV 1 and HIV 2 and PCR COVID-19 were negative. The abdominal ultrasound revealed normal appearing liver (141mm) without evidence of intrahepatic biliary dilatation. The gallbladder wall was thickened measuring approximatively 7.7mm without evidence of gallstones. Murphy´s sonographic sign was positive. No pericholecystic fluid collection (Figure 1).

**Diagnosis:** the presumptive diagnosis were acute hepatitis and acute cholecystitis post vaccination. According to the findings, the results were consistent with acute acalculous cholecystitis following COVID-19 vaccination.

**Therapeutic interventions:** paracetamol infusion (1g/day), diclofenac ampule (75mg/day) and ringer lactate (1L/day) were administrated intravenously.

**Follow-up and outcome of interventions:** the patient did not respond to the treatment. Antibiotics were added: cefuroxime tablets 500mgr (bd x5/7), rabeprazole tablets 20mgr (odx2/52), livolin tablets (odx2/52). The patient had an excellent response to antibiotics with a regression of clinical symptoms but with elevated liver enzyme after two weeks of follow-up (Table 1).

**Table 1:** evolution of blood abnormal parameters during 10 days of hospitalization and two weeks of follow-up testing

	Hematological parameters		Biochemistry (liver test+)				
Follow-up days	Platelet	White blood cell count	AST/SGOT	Alkaline phosphatase	ALT/SGPT	GGT	C-reactive protein
Day 1 (11/11/2021)	79(140-440)10*g/L	2.83(4-11)10*g/L	493.9(<38)IU/L	106(30-120)IU/L	244.7(0-40)U/L	85(5-40) U/L	148.2(<5)mg/L
Day 2 (12/11/2021)	71(140-440) 10*g/L	1.95(4-11) 10*g/L	619.4(0-40)IU/L	99(30-120)IU/L	295.7(0-40)U/L	93(11-50) U/L	
Day 3 (13/11/2021)			418.1(0-40)IU/L	115(30-120)IU/L	288.1(0-40) U/L	134(11-50) U/L	
Day 4 (15/11/2021)	175(140-440) 10*g/L	4.19(4-11) 10*g/L	268.1(0-40)IU/L	193(30-120)IU/L	292.6(0-40) U/L	293(11-50) U/L	
Day 5 (17/11/2021)	236(140-440) 10*g/L	5.15(4-11) 10*g/L					19.95(0.0-7)mg/L
Day 6 (20/11/2021)			65.3 (0-40)IU/L	188 (30-120)IU/L	173.8 (0-40) U/L	211(11-50) U/L	
Day 7 (26/11/2021)	236(140-440) 10*g/L	4.37(4-11) 10*g/L	30.4 (0-40)IU/L	187 (30-120)IU/L	88.6 (0-40) U/L	180(11-50) U/L	
Day 8 (01/12/2021)			32.5(0-40)IU/L	147(30-120)IU/L	70(0-40) U/L	142(11-50) U/L	

AST: aspartate aminotransferase; SGOT: serum glutamic oxaloacetic transaminase; ALT: alanine aminotransferase; SGPT: serum glutamic pyruvic transaminase; GGT: gamma-glutamyl transferase. The table shows that after 4 days of treatment the platelet and blood white cells count come to the normal range, liver enzyme alkaline phosphatase, ALT and GGT still be high after two weeks of follow-up.

**Informed consent:** the patient provided a full consent after oral explanation of our intention of publishing his case.

## Discussion

Acute acalculous cholecystitis is a rare disease and difficult to diagnose. It represents 10% of acute cholecystitis and its mortality is higher than lithiasic cholecystitis [6,7]. It has been reported to old patients with no underlying diseases suffering from COVID-19. Mattone *et al*. reported a case of acute acalculous cholecystitis to a 66-year-old man, ex-smoker with no medication and story of genetic diseases tested positive to COVID-19 and treated by percutaneous transhepatic gallbladder drainage followed by laparoscopic cholecystectomy due to no clinical improvement [8]. Ying *et al*. reported also an acute cholecystitis to a 68- year-old female with no underlying condition tested positive to COVID-19 with elevated blood levels for C-reactive protein (33.7 mg/L; normal range, 0-10 mg/L) [9]. In these two cases the acute acalculous cholecystitis is likely to be caused by COVID-19 infection. In the present case, contrary to Mattone *et al*. [8] and Ying *et al*. [9], the subject was a young 29-year-old man, tested negative to COVID-19 PCR test, with no underlying conditions, diagnosed with acute acalculous cholecystitis following COVID-19 vaccine inoculation. In addition, about 7.98 million doses of the Janssen COVID-19 vaccine that were administrated from March 2^nd^ to April 21, 2021 in United States of America, no case of acute acalculous cholecystitis among vaccine recipients was reported by the Vaccine Adverse Event Reporting System (VAERS) [5]. We suppose that acute acalculous cholecystitis is likely to be a new adverse event of Janssen COVID-19 vaccine.

## Conclusion

Acute acalculous cholecystitis is likely to be induced by COVID-19 infection in rare cases reported in elderly subjects. We have reported a case of acute acalculous cholecystitis in a young subject with no COVID-19 infection following Janssen COVID-19 vaccine inoculation. We think that this vaccine in rare case could induce the disease and therefore could be a new adverse event of this vaccine.
